# Supplementary material for: Exome Analysis Reveals Novel Missense and Deletion Variants in the CC2D2A Gene as Causative of Joubert Syndrome
Source: Genes (Basel). 2023 Mar 28;14(4):810. doi: 10.3390/genes14040810 (PMC10137517; doi:10.3390/genes14040810)
Supplement: Supplementary file 1 [file genes-14-00810-s001.zip › genes-2269059-supplementary.pdf]

Supplementary Tables

Table S1. Joubert Syndrome gene panel according to Human Phenotype Ontology. * Coverage of ≥82% and ≤ 97% with a target read depth 20X.						
AHI1	ARL13B	ARL3	ARMC9*	B9D1	B9D2	CC2D2A*
CEP104	CEP120	CEP290*	CEP41	CPLANE1	CSPP1*	FAM149B1
HYLS1	INPP5E	KIAA0556	KIAA0586*	KIF7	MKS1	NPHP1*
OFD1	PDE6D	PIBF1*	RPGRIP1L	SUFU	TCTN1	TCTN2
TCTN3	TMEM107	TMEM138	TMEM216	TMEM231	TMEM237	TMEM67*
TTC21B	ZNF423					

Table S2. Primers used in Sanger Sequencing

Primer name	Primers (5'-3')	Amplicon size (bp)
CC2D2A-ex30-F	GGGATTGGCTTTGAATGCCAG	409
CC2D2A-ex30-R	GAAGGCTCTGTTTGTGTAGGAAA	

Table S3. Primers used in qPCR experiments

Primer name	Primers (5'-3')	Amplicon size (bp)
CC2D2A-ex29-F	CTGGCTGGGATGTGTGAA	87
CC2D2A-ex29-R	CCTGCTCTCATGGACCTCTAA	
CC2D2A-ex30-F	CTGGTTCCTGGAGAGTCCAT	93
CC2D2A-ex30-R	CCTAGCAAGCATTTCTTGTGTT	
CC2D2A-ex31-F	TCGTCAGTGCCTTACAACAGT	87
CC2D2A-ex31-R	TCCTGAGGAGGGTTTAAAGGT	
CC2D2A-ex32-F	AGGAACTGGTGGCTCGATA	87
CC2D2A-ex32-R	ATGTGCTCCAGAGGTCACAGA	
GAPDH-F	CTGCACCACCAACTGCTT	87
GAPDH-R	CCCAGCTCTCATACCATGAGT	
